# Pregnancy Outcomes in Women with Long-Duration Type 1 Diabetes—25 Years of Experience

**DOI:** 10.3390/jcm9103223

**Published:** 2020-10-08

**Authors:** Ewa Wender-Ozegowska, Paweł Gutaj, Urszula Mantaj, Jakub Kornacki, Stefan Ozegowski, Agnieszka Zawiejska

**Affiliations:** 1 Department of Reproduction, Poznan University of Medical Sciences, Polna 33, 60-525 Poznań, Poland; pgutaj@o2.pl (P.G.); urszula.mantaj@gmail.com (U.M.); kuba.kornacki@wp.pl (J.K.); agazaw@post.pl (A.Z.); 2 2nd Department of Cardiology, Poznań University of Medical Sciences, 28 Czerwca 1956r. nr 194, 61-485 Poznań, Poland; stefano@post.pl

**Keywords:** diabetes, hypertension, pregnancy, proteinuria, type 1 diabetes, vasculopathy

## Abstract

Aims: Our study aimed to examine the pregnancy outcomes (maternal and fetal) concerning different models of antenatal care across a period of over 25 years (1993–2018) in 459 women with type 1 diabetes. Data from patients with a history of the condition lasting at least 15 years were considered eligible for analysis. Methods: The study group was divided into three cohorts based on the different models of treatment used in Poznan University Hospital, Poland: 1993–2000 (cohort I, *n* = 91), 2001–2005 (cohort II, *n* = 83), 2006–2018 (cohort III, *n* = 284). To identify predictors for the selected dichotomous outcomes, we calculated the risks for fetal or maternal complications as dependent variables for cohorts II and III against cohort I, using multivariate logistic regression analysis. Results: The mean gestational age was 36.8 ± 2.4 weeks in the total cohort. The percentages of deliveries before the 33rd and the 37th weeks was high. We observed a decreasing percentage during the following periods, from 41.5% in the first period to 30.4% in the third group. There was a tendency for newborn weight to show a gradual increase across three time periods (2850, 3189, 3321 g, *p* < 0.0001). In the last period, we noticed significantly more newborns delivered after 36 weeks with a weight above 4000 g and below 2500 g. Caesarean section was performed in 88% of patients from the whole group, but in the subsequent periods this number visibly decreased (from 97.6%, 86.7%, to 71%, *p* = 0.001). The number of emergency caesarean sections was lowest in the third period (27.5%, 16.7%, 11.2%, *p* = 0.006). We observed a decreasing number of “small for gestational age” newborns (SGA) in consecutive periods of treatment (from 24.4% to 8.7%, *p* = 0.002), but also a higher percentage of “large for gestational age” (LGA) newborns (from 6.1% to 21.6%, *p* = 0.001). Modification of treatment might be associated with the gradual reduction of SGA rates (cohort I 3.6%, cohort III 2.3% *p* < 0.0005). Conclusions: Strict glycemic and blood pressure control from the very beginning of pregnancy, as well as modern fetal surveillance techniques, may contribute to the improvement of perinatal outcomes in women with long-duration type 1 diabetes.

## 1. Introduction

The impact of long-duration diabetes (LDD) on pregnancy has been intensively researched for many years. The results are often inconsistent, primarily due to the heterogeneity of study groups, including diverse metabolic control, different diagnostic criteria for perinatal complications and different classification systems [1,2].

The duration of diabetes is the main risk factor for the development of diabetic vasculopathy. Almost all patients with type 1 diabetes (T1DM) develop changes in the eye after 20 years [3]. Studies show a relationship between diabetic retinopathy and perinatal outcome. McElvy et al. showed that the presence of retinopathy was significantly associated with lower mean birth weight and a higher rate of “small for gestational age” infants [4]. Klein et al. reported that the severity of retinopathy was the only variable that significantly predicted adverse perinatal outcome [5]. Nephropathy, which occurs in 20%–30% of patients with type 1 diabetes, also occurs in 5%–10% of pregnancies complicated by pregestational diabetes (PGDM) [6,7]. Pregnancy outcome is related to pre-pregnancy renal function [8,9]. Proteinuria may develop for the first time during pregnancy and may indicate pre-eclampsia, which develops in 30%–65% of pregnancies with severe diabetic nephropathy, i.e., with daily proteinuria above 1 g/day at the beginning of pregnancy [8,9,10].

Pregnancy preparation should be multifaceted, with strict metabolic and blood pressure control. Two studies showed that the use of antihypertensive therapy (angiotensin-converting enzyme inhibitors, ACE) for six months and strict metabolic control three months before diabetic pregnancy significantly reduced the severity of proteinuria in early pregnancy. Moreover, the renoprotective effect persisted in the later course of pregnancy [11,12]. Carr et al. observed significantly higher proteinuria and higher serum creatinine levels, and also indicated the need to complete the pregnancy before 32 weeks of gestation. This was associated with the deterioration of renal function in pregnant women with high values of mean arterial pressure [13]. In patients with advanced nephropathy in early pregnancy, a rapid progression to end-stage renal disease and the need for dialysis was observed. Noteworthy too is that in these women, worse metabolic control and the development of additional complications were also seen [8,13].

Due to an increased incidence of type 1 diabetes, more women need to consider the effects of long-duration diabetes on pregnancy outcomes. In recent decades, outcomes for pregnant women with diabetes have improved due to improved education and better treatments. However, comprehensive reports on pregnancy outcomes of patients with long-duration type 1 diabetes patients are still too few.

Our study aimed to examine the pregnancy outcomes (maternal and fetal) concerning different models of antenatal care across a period of over 25 years (1993–2018) and to investigate risk factors for perinatal complications among pregnant women with long-duration diabetes treated in three periods with different models of treatment.

## 2. Experimental Section

### 2.1. Subjects

We present a retrospective analysis of maternal and fetal outcomes in pregnant women with type 1 diabetes treated between 1993 and 2018. Data of patients with a history of the condition lasting at least 15 years were considered eligible for the analysis. Obstetric data of 1153 patients with singleton pregnancies treated in Poznań University Hospital (PUH), the leading tertiary center for pregnant women with diabetes in Poland which covers a population of 3.4 million people, were analyzed. From the whole group, we selected 459 (39.8%) patients with diabetes lasting at least 15 years (LDD).

The 15 years of diabetes duration as a criterion for recruiting our patients was based on the data showing that after this time the risk for vascular complications (nephropathy, retinopathy) rises visibly in several studied groups [14].

The most recent childbirth data were included. Data lacking information concerning fetal outcome were excluded. Finally, we included 450 (39.02%) mother/infant pairs. Women with overt proteinuria, arterial hypertension (AH) diagnosed before pregnancy and retinal lesions were classified as having vascular complications (VC). Among women whose pre-pregnancy blood pressure (BP) was unknown, the diagnosis of AH was based on the presence of hypertension before 20 weeks of gestation, defined as either systolic BP of at least 140 mm Hg or diastolic BP of at least 90 mm Hg on at least two occasions measured at least 4 h apart. Gestational hypertension (GH) was defined as a BP higher than 140/90 mm Hg measured on two separate occasions, more than 4 h apart, without the presence of protein in the urine and diagnosed after 20 weeks of gestation. We diagnosed pre-eclampsia in a previously normotensive woman with a new onset of hypertension on at least two occasions at least four hours apart and proteinuria after 20 weeks of gestation. In the absence of proteinuria, the diagnosis we made of new-onset hypertension was accompanied by symptoms of significant end-organ dysfunction. Pre-eclampsia in patients with AH and proteinuria we defined as a worsening of hypertension (≥15 mmHg increase in systolic and or diastolic BP) with a proteinuria level of ≥ 3 g/24 h after 20 gestational weeks or in the presence of one of the following events: thrombocytopenia and/or elevated serum aminotransferases concentration.

Diabetic nephropathy was defined as total protein excretion in urine ≥ 0.3 g/24 h. Routinely, patients with diabetic proliferative retinopathy had laser photocoagulation treatment before or during pregnancy.

### 2.2. Obstetric Procedure

Patients with type 1 diabetes were referred to the PUH as soon as the pregnancy had been diagnosed or when the patient first reported her pregnancy. The gestational age was confirmed or corrected by sonography in the first trimester. Patients without complications had at least three planned short-stay hospital admissions during pregnancy: in the first trimester, in mid-pregnancy (20th–24th weeks) and near delivery (34th–35th weeks). Patients who required more vigilant surveillance were admitted more frequently and the last hospitalization was no later than in the 34th week. Between hospital admissions, patients were referred for check-ups in the outpatient clinic every two weeks. “Small for gestational age” fetuses (SGA) were defined as birth weight lower than the tenth percentile and “large for gestational age” (LGA) as birth weight greater than the ninetieth, using age- and sex-specific regional growth charts [15].

### 2.3. Monitoring of Laboratory Measurements

All women were treated with intensive insulin therapy (MDI) during both periods of observation. During the third period (2006–2018), women were treated either with MDI or with continuous subcutaneous insulin infusion (CSII). The patients measured their fasting blood glucose and all pre- and postprandial values every week and took 5–6 daily glucose measurements on other days.

Blood samples for other analysis were taken after overnight fasting and immediately transported to the laboratory of the PUH. HbA1C (Haemoglobin A1C) in whole blood was determined using the turbidimetric inhibition immunoassay (TINIA), Tina-quant Hemoglobin A1c II test in a Cobas c311 analyzer (Roche Diagnostics, Rotkreuz, Switzerland). The normal range is 29–42 mmol/mol (4.8%–6.0%) for a non-pregnant population. The analysis method of HbA1C did not change during the study periods. We used three HbA1C values: the first was measured in the I trimester or first admission, the second was measured in the II trimester between 18 + 0 and 22 + 0 gestational weeks (GW) and the last before delivery. If two HbA1C values had been measured in any period, we used the average value.

### 2.4. Collection of Maternal and Obstetric Data

Pre-pregnancy weight and patient height were obtained from the department database. Data on pregnancy planning, onset of diabetes, duration, complications, antihypertensive medication and delivery modes were all collected from the same database and hospital records. The second highest systolic and diastolic BP values in each trimester were recorded.

Data on gestational age at birth, birth weight, APGAR scores, neonatal status and arterial umbilical pH value were obtained from patient records. Umbilical artery blood samples were taken routinely and analyzed for pH using Ciba Corning (Bayer Diagnostics, Fernwald, Germany).

We analyzed models of treatment in three periods with actual, visibly changed routine antenatal procedures in the PUH. These procedures followed the Polish Diabetes Association (PDA) and Polish Gynecological Association (PGA) standards for women with PGDM. We also adopted recommendations for patients with LDD published by a Danish group [2]:

- Cohort I (*n* = 91): the first period between 1993 and 2000 (target blood pressure—TBP) ≤ 140/90, antihypertensive treatment in pregnancy (AHTP) not related to the stage of proteinuria, HbA1C in pregnancy < 53 mmol/mol (<7.0%), target fasting glycemia (FG) < 5.0 mmol/L, 1 h postprandial (PP) < 7.8 mmol/L, 2 h PP < 6.7 mmol/L).
- Cohort II (*n* = 83): the second period between 2001 and 2005 (TBP < 135/85, ACE inhibitors before pregnancy, HbA1C < 53 mmol/mol (<7.0%), AHTP if proteinuria > 1.9 g/24 h, target FG < 5.0 mmol/L, 1 h PP < 7.8 mmol/L, 2 h PP < 6.7 mmol/L).
- Cohort III (*n* = 284): the third period between 2006 and 2018 (TBP < 135/85, ACE inhibitors before pregnancy, HbA1C < 43 mmol/mol (<6.1%), AHTP if proteinuria > 0.3 g/24 h, target FG 3.3–5.0 mmol/L, PP < 6.7 mmol/L); auxiliary doppler monitoring to support a decision about timing delivery. Absent end-diastolic flow (AEDF) in the umbilical artery at or after 34 weeks or reversed end-diastolic flow in the umbilical artery at or after 32 weeks or a cerebral umbilical ratio (CUR) below 5th percentile at or after 37 weeks were the indications for immediate delivery.

Only 3% of patients in the last period of observation were treated with aspirin from the 8th week of pregnancy.

We analyzed the fetal and maternal results in the studied groups of patients and also calculated the risks for selected maternal or adverse fetal outcomes using data from our three consecutive cohorts; results of patients from the first period (1993–2000, cohort I) served as a baseline cohort.

### 2.5. Statistics

Statistical analyses were performed using Statistica software version 13.1 (Statsoft, Cracow, Poland) and SPSS Statistics 22.0 (IBM corp. Armonk, New York NY, U.S.A.). Continuous variables were analyzed with the Mann–Whitney U test or paired-samples t-test, with ANOVA followed by Tukey’s test, or with the Kruskal–Wallis test and Bonferroni correction to allow for multiple testing. The Χ^2^ and Fisher’s exact tests were used in the analysis of categorical variables.

Results of a post-hoc analysis performed against the prevalence of fetal and maternal adverse outcomes in the baseline cohort, which presented a calculated power above 70%, were selected for further analysis. To identify predictors for the selected dichotomous outcomes, we calculated the risks for fetal or maternal complications as dependent variables for cohort II and cohort III against cohort I, using multivariate logistic regression analysis.

## 3. Results

### 3.1. Maternal Characteristics

We present a retrospective analysis of 450 consecutive diabetic pregnancies referred to the tertiary-level of care unit for treatment between 1993 and 2018 due to T1DM with a history of the condition for longer than 15 years.

The number of patients with LDD transferred to our unit increased substantially across the study periods, as did the maternal age throughout the analyzed periods, which translated into a significantly longer history of diabetes as the age at the onset of the condition remained similar among all three subgroups (Table 1). A significant difference was seen in BMI (body mass index), however the percentage of overweight and obese patients was similar in all groups. Women from the latest cohort were also referred to tertiary-level antenatal care significantly earlier, usually in the first trimester.

We also noted a trend of a decreasing number of patients with AH in cohort II and cohort III subgroups, compared to cohort I. The percentage of patients with GH was highest in the second period of observation.

Our study group showed a predominantly poor metabolic control in the periconceptional period, with a significant improvement in HbA1C in the second half of pregnancy.

Total protein excretion in urine differed significantly among studied cohorts. The first-trimester daily protein loss reached the highest value in cohort I, but in the third trimester, the highest loss was noted in cohort III. The renal function measured as a serum creatinine remained similar among all three cohorts.

In cohort III, we observed the highest percentage of patients without vascular complications (VC), despite the longest duration of the condition in this group (Table 2). The highest frequency of VC was observed in cohort II, with 26.2% of all patients manifesting retinopathy and nephropathy already at the beginning of pregnancy, up to 36% of the baseline cohort. Two patients became pregnant after renal transplantation and two after myocardial infarctions. In each case, the pregnancy had a successful outcome.

There is no difference between the subgroups without VC regarding their age, duration of diabetes, onset of the condition, initial management in pregnancy and HbA1C in the first trimester (Table 3). Nevertheless, when we take a look at the subgroups with retinopathy or nephropathy, we see that in the latest cohort women with VC were significantly older and their diabetes had a longer duration. However, they were under the care of our department noticeably earlier.

### 3.2. Perinatal Outcome

Over these 25 years, we noticed 37 (8.2%) spontaneous miscarriages, the number decreasing from 9.9% in the first period to 7.6% in the last. None of the patients included in the study opted for pregnancy termination. Major malformations were diagnosed in 23 newborns, which account for around 3.6% of the whole cohort.

The mean gestational age at delivery was 36.8 ± 2.4 weeks (Table 4). Premature delivery was the most common perinatal complication in the cohort. The percentages of deliveries before the 33rd and the 37th GW remained high, with a trend of reduced prevalence from 41.5% in the baseline cohort to 25.5% in the third cohort.

We observed in the consecutive periods a tendency towards larger mean newborn weight and this was significantly different between the groups (2850, 3189, 3321 g, *p* < 0.0001). In the latest period of observation, we noticed significantly more newborns delivered at term with a weight above 4000 g and with a weight below 2500 g. Caesarean section was the predominant mode of delivery in our study group, with a statistically significant decrease across the cohorts from 97.6% in cohort I, to 86.7% in cohort II and 71% in cohort III (*p* < 0.001). A similar trend was noted for emergency caesarean sections (27.5%, 16.7%, 11.2%, respectively, *p* < 0.006). APGAR score below 8 was significantly more often given to the newborns in the baseline cohort. Umbilical artery pH values in the umbilical artery did not differ among the periods of observation. We registered three cases of early neonatal death in cohort I and two such cases in cohort II. Four of them occurred in patients with class RF (proliferative retinopathy and nephropathy) because of prematurity (26–34 weeks) and one in class F (nephropathy), because of heart malformation.

In patients without VC, we found no differences in the neonatal outcomes among the subgroups (Table 5). In the retinopathy-only group, we observed a trend towards higher birth weights, fewer SGA cases and a significantly improved newborn condition measured as 1 min APGAR score. Similar trends were noted across the study periods in the subgroup with isolated nephropathy. In the patients with combined nephropathy and retinopathy, we noticed a significant increase in pregnancy duration accompanied by a significant increase in the birth weight and APGAR score across the cohorts. In these patients, we also noted a trend towards a reduced number of early neonatal deaths.

Table 6 presents the results of the post-hoc analysis performed to show the prevalence of fetal and maternal adverse outcomes in cohort II and cohort III against cohort I. Generally, the logistic regression analysis indicated that the most recent cohort had a significantly reduced risk for all perinatal complications, except for neonatal macrosomia, for which the risk increased non-significantly (OR 2.16, 95% CI: 0.55; 12.44). Several maternal characteristics had a significant impact on the perinatal outcomes, irrespective of the period of treatment. Particularly, arterial hypertension was significantly related to an increased risk of premature delivery and emergency caesarean section (OR 2.1; 95% CI: 1.13; 3.92 and OR 2.26; 95% CI: 1.08; 4.74, respectively).

Importantly, we identified chronic arterial hypertension as a protective factor, significantly reducing the risk of developing pre-eclampsia later in pregnancy (Table 7).

Parameters of renal function in early pregnancy were available in a limited number of records. Therefore, we performed a separate analysis to identify whether renal function measured as GFR, serum creatinine level or daily proteinuria in the first trimester was associated with maternal or fetal adverse outcomes. The results are summarized in Table 8.

Overall, we noticed that the patients from more recent cohorts were significantly more likely to have early pregnancy GFR above 120 mL/min/1.73 m^2^ (OR 8.7 (2.2; 34.5) *p* = 0.002 for cohort II, OR 15.2 (4.5; 51.4) *p* < 0.001 for cohort III) and less likely to express overt proteinuria (OR 0.10 (0.02; 0.53) *p* = 0.007 for cohort III).

## 4. Discussion

In this retrospective cohort of pregnancies with LDD, we aimed to analyze maternal and fetal outcomes concerning the period of observation. The main strength of the study is the large, well-characterized cohort, including only patients with LDD and treated in one tertiary center. Authors have been responsible for the treatment, the protocols during the study period as well as for the collection of data. Limitations include the observational nature of the study as well as certain changes in general obstetric and neonatal management during the long study period. We also had to cope with the clinical reality in which the vast majority of patients did not plan their pregnancies, which could cause a lack of some of the data.

During the time of analysis, several aspects of care in diabetes changed, especially the understanding of complications in pregnancy with LDD. In past studies, antihypertensive treatment during pregnancy for mild chronic hypertension had not been proven to have an impact on pregnancy complications, such as PE or preterm delivery. Over the years, we have modified the care for diabetic pregnancies, and especially for women with vasculopathy [9,16,17,18]. Our attention was given to the much stricter control of BP from the beginning of pregnancy and lower targets for glycemia. The PDA has recommended, since the year 2000, to all patients with diabetes, ACE inhibitors or angiotensin II receptor blockers in the case of hypertension or the presence of microalbuminuria [3,19].

However, in the analyzed groups, treated in different periods, we observed a tendency to become pregnant later in life. The number of patients treated per year in the last period was almost doubled in comparison to the first one. Because the age of patients when they developed diabetes was not significantly different, patients in cohort III presented significantly longer duration of the condition and were therefore predisposed to more severe complications, both before as well as during the pregnancy. Very similar observations were presented by Klemetti et al. [20]. Similarly to Klemetti et al., we noticed a tendency for higher BMI in consecutive groups, but the number of overweight and obese women were not significantly different between them.

The fundamental goal for women with diabetes is to plan their pregnancy [21,22]. However, in our cohort the percentage of women attending prenatal counseling did not exceed 25% and the beginning of observation started mainly after the first trimester of pregnancy. In the last period, the average beginning of care was in the first trimester and in that group HbA1C was lower than in the others. During pregnancy, patients treated in the latest period achieved, almost as expected, control of glycemia. In other studies, patients with LDD, microalbuminuria or nephropathy also present much higher HbA1C at the beginning of pregnancy than recommended [18,20]. Serum creatinine was used as an index of renal function. We found stable serum creatinine levels during pregnancy for analyzed women in all periods.

Success in diabetic pregnancy relates not only to glycemia but also several other parameters, especially well-controlled blood pressure [23,24]. We found a visible difference between patients in all groups. Even though patients in cohort III had the longest diabetes duration, cohort III had the highest percentage of patients without vascular complications and the lowest number with nephropathy and retinopathy. The percentage of patients with nephropathy and retinopathy declined from 35% to 21%, and those with nephropathy only almost halved from 7.6% to 3.2%. We did notice, however, an increased percentage of proliferative retinopathy (from 14.3 to 20.1) in cohort III. Damm et al. reported that in Denmark, the prevalence of diabetic nephropathy had declined by half, from 5% in the late 1990s to 2.5% in 2013 [23]. The decline in the prevalence of diabetic nephropathy and the rise in the prevalence of women with LDD without complications is in line with a general trend over recent years in diabetes. It may be explained by improved glycemic control and more frequent use of ACE inhibitors [19,25]. None of the analyzed parameters in patients without complications differ between the subgroups. Still, in women with nephropathy and retinopathy observed in the last period, the duration of diabetes was the longest and patients were the oldest. However, they were treated from the earliest week of pregnancy.

Our study shows that we have not improved results concerning spontaneous miscarriages or malformation ratios, because a low percentage of our patients plan their pregnancy and the mean HbA1C in the first trimester was far from recommended. However, it is well known that counseling is essential for a successful outcome of pregnancy in women with diabetes [26]. We have noticed an apparent reduction in the number of premature deliveries, very small babies, neonatal death and the frequency of emergency caesarean sections in the last period of treatment, especially in the group of patients with vascular complications.

The total percentage of SGA babies fell in analyzed periods from 24.4% to 8.7% (*p* < 0002). In consecutive periods, we observed a reduction in the number of deliveries before 32 weeks, but this difference was not significant. In subsequent periods we observed a higher percentage of LGA babies, which might be connected to increased maternal weight in the last period [27].

All patients with long duration of diabetes remain under very tight control in our center and are hospitalized, mainly, at around 34 weeks of pregnancy or even earlier, especially when problems with hypertension or increased proteinuria exist. We have not found any intrauterine death in our cohort after 22 weeks of pregnancy and just 1.1% of neonatal death. In our opinion, this was the result of the regular care given to this group and also thanks to the policy in our center, i.e., early hospitalization of patients, especially with nephropathy and hypertension. Nielsen et al. presented a comparison of results of patients with diabetes with vascular complications concerning standards of treatment and also showed a significant reduction in the risk of adverse fetal outcome [28].

It is known that AH, pregestational microalbuminuria or renal disease, poor metabolic control and long duration of diabetes increase the risk of PE. However, some studies do not confirm this [8,29]. In our study, we have not observed a significant number of PE patients, but our data show that many patients with nephropathy presented increased proteinuria during pregnancy and also in the last period of treatment. Nevertheless, in our opinion, the latest model of treatment with strict antihypertensive control started early in pregnancy, and relatively good metabolic control, protected them against the development of pre-eclampsia, which was also confirmed in different studies [23,29]. The role of hyperglycemia in mediating PE is unclear [30].

A consistent increase in preterm delivery with rates exceeding 50% and fetal growth restriction (15% of cases) have been reported in American and European services over the last 25 years [29]. Our results from the last 25 years confirmed that intensification of treatment resulted in reduction of fetal complications. Besides the model of treatment, the significant influence on prematurity and emergency deliveries had the following baseline maternal parameters: age at diabetes diagnosis, maternal hyperglycemia and the presence of AH. Lower and higher newborn weight was in an opposite way related to maternal weight and glycemia.

The analysis of maternal baseline status and maternal outcomes for the whole study group revealed that the standards of treatment had a significant influence on the maternal outcome and, surprisingly, the presence of AH had a positive effect. Our explanation for this observation is that in the first and second period of our observation, defined hypertension required intensive antihypertensive treatment from the beginning of pregnancy, that was not introduced in women with slightly increased BP. We have not confirmed data presented by other authors, that glycemic control and pre-pregnancy BMI have a significant impact on pre-eclampsia in women with T1DM [31,32,33]. This might be due to the relatively good glycemic control shown by a high rate of women in the early second and third trimester and also thanks to more precise methods estimating fetal condition, such as fetal doppler ultrasound. Moreover, a reduced number of patients with VC in more recent cohorts also suggest that the population is at lower risk of placental insufficiency which is the major risk for pre-eclampsia and fetal demise.

To summarize, our results confirm the hypothesis that improved antenatal blood pressure control improves neonatal and maternal outcomes. Although our retrospective study design cannot support this assumption with certainty, we believe that we have presented compelling evidence to support that position. Firstly, our results are bolstered by strong data presented by other authors that have also modified the standards of treatment of pregnant women with LDD. Secondly, we collected a representative group of patients treated in one tertiary center during the analyzed period.

## 5. Conclusions

Strict glycemic and blood pressure control from the very beginning of pregnancy and modern fetal surveillance techniques may reduce adverse perinatal outcomes for mothers with long-duration type 1 diabetes and their newborns.

## Figures and Tables

**Table 1:** Maternal characteristics of 450 women with long-duration diabetes in three periods of observation between 1993 and 2018.

Parameter	1993–2000 I	2001–2005 II	2006–2018 III	All	*p*-Value
Number of patients, *n* (%)	91 (20.2)	83 (18.4)	276 (61.3)	450 (100.0)	
Number of patients/years, *n*	11.4	16.6	21.2	18.0	
Age, years (IQR) *	26.0 (22.5–30.5)	26.0 (23.0–29.0)	28.0 (25.0–31.7)	27.0 (24.0–31.0)	*p* < 0.01
Duration of diabetes, years (IQR) *	16.5 (16.5–20.5)	15.5 (12.0–20.0)	18.0 (14.0–22.0)	17.6 (13.0–21.0)	*p* < 0.005
Age at time of diagnosis of DM, years (IQR) *	9.0 (6.0–13.0)	9,0 (8.0–14.0)	9.0 (6.0–12.0)	9.0 (6.0–13.0)	*p* = 0.39
Patient’s height, cm (IQR) *	162.0 (158.0–164.0)	164.0 (160.0–168.0)	164.0 (160.0–169.0)	164.0 (160.0–168.0)	*p* < 0.02
Patient’s weight, kg (IQR) *	58.0 (54.0–64.0)	62.0 (55.0–68.0)	63.3 (57.0–70.8)	62.0 (56.0–69.0)	*p* < 0.002
BMI at the beginning of pregnancy, kg/m2 (IQR) *	22.1 (20.8–24.4)	22.2 (20.5–25.1)	23.3 (21.1–26.1)	22.9 (20.9–25.6)	*p* < 0.02
BMI at the end, kg/m2 (IQR) *	26.6 (24.5–28.6)	26.7 (25.2–29.3)	28.0 (25.4–31.0)	27.0 (24.4–30.5)	26.6 (24.5–28.6)
Number of patients with BMI 25–30 kg/m2, *n* (%) **	11 (12.1)	16 (19.3)	66 (23.9)	93 (20.7)	Gr.I vs. G.II *p* = 0.19 Gr.I vs. Gr.III *p* < 0.02 Gr.II vs. Gr.III *p* = 0.41
Number of patients with BMI > 30 kg/m^2^, *n* (%) **	2 (2.2)	5 (6.0)	19 (6.9)	26 (5.8)	Gr.I vs. Gr.II *p* = 0.24 Gr.II vs. Gr.III *p* = 0.81 Gr.I vs. Gr.III *p* = 0.12
Planning the pregnancy, *n* (%) **	18 (19.6)	14 (16.7)	68 (24.3)	100 (26.3)	Gr.I vs. Gr.II *p* = 0.61 Gr.II vs. Gr.III *p* = 0.14 Gr.I vs. Gr.III *p* = 0.34
Beginning of observation, week (IQR) *	11.0 (8.0–16.0)	8.0 (6.0–13.0)	8.0 (7.0–9.0)	8.0 (7.0–11.0)	*p* < 0.0001
Number of patients with arterial hypertension, *n* (%) **	23 (23.3)	11 (13.3)	46 (16.7)	80 (17.8)	Gr.I vs. Gr.II *p* < 0.05 Gr.II vs. Gr.III *p* = 0.25 Gr.I vs. Gr.III *p* = 0.07
Number of patients with gestational hypertension, *n* (%) **	1 (1.1)	16 (19.3)	12 (4.4)	29 (5.8)	Gr.I vs. Gr.II *p* < 0.0002 Gr.II vs. Gr.III *p* < 0.00001 Gr.I vs Gr.III *p* = 0.14
Beginning of gestational hypertension, weeks (IQR) *	27.0 (27.0–27.0)	32.0 (25.0–33.5)	31.0 (26.5–33.5)	31.0 (26.0–33.0)	Gr.I vs. Gr.II *p* < 0.05 Gr.II vs. Gr.III *p* = 0.07
No of patients with PET, *n* (%) **	0 (0)	0 (0)	11 (4.0)	11 (2.44)	Gr.I vs. Gr.III *p* = 0.053 Gr.II vs. Gr.III *p* = 0.06
HbA1C I trim., mol/mol (IQR) *	61.7 (50.8–72.7)	59.6 (46.4–78.1)	59.6 (47.5–69.4)	58.5 (47.5–70.5)	*p* = 0.14
HbA1C I trim., % (IQR) *	7.8 (6.8–8.8)	7.6 (6.4–9.3)	7.6 (6.5–8.5)	7.5 (6.5–8.6)	*p* = 0.14
HbA1C II trim., mol/mol (IQR) *	44.3 (34.4–61.7)	48.6 (42.1–57.4)	42.1 (34.4–47.5)	42.1 (34.4–50.8)	*p* < 0.001
HbA1C II trim., % (IQR) *	6.2 (5.3–7.8)	6.6 (6.0–7.4)	5.9 (5.3–6.5)	6.0 (5.3–6.8)	*p* < 0.001
HbA1C III trim., mol/mol, (IQR) *	48.6 (36.6–59.6)	46.8 (46.4–53.0)	44.4 (43.2–49.7)	44.3 (37.7–51.9)	*p* = 0.08
HbA1C III trim (last before delivery), % (IQR) *	6.6 (5.5–7.6)	6.4 (5.7–7.0)	6.1 (5.6–6.7)	6.2 (5.6–6.9)	*p* = 0.09
Diurnal protein loss I trim., g/24 h (IQR) *	0.8 (0.6–1.6)	1.8 (0.7–2.5)	0.14 (0.1–0.3)	0.1 (0.0–0.3)	*p* < 0.001
Diurnal protein loss II trim., g/24 h (IQR) *	1.5 (0.5–2.6)	0.6 (0.5–2.6)	0.2 (0.1–0.4)	0.2 (1.1–0.6)	*p* < 0.05
Diurnal protein loss III trim., g/24 h (IQR) *	2.6 (0.6–4.7)	0.6 (0.2–3.0)	0.4 (0.2–1.2)	0.4 (0.2–1.3)	*p* < 0.01
Creatinine I trim., mg/dL (IQR) *	0.8 (0.7–0.9)	0.7 (0.5–0.8	0.6 (0.5–0.7)	0.6 (0.6–0.7)	*p* < 0.0001
Creatinine II trim., mg/dL (IQR) *	0.8 (0.6–1.0)	0.7 (0.6–0.7)	0.5 (0.5–0.7)	0.6 (0.5–0.8)	*p* < 0.0001
Creatinine III trim., mg/dL (IQR) *	0.8 (0.7–1.0)	0.6 (0.6–0.8)	0.7 (0.6–0.8)	0.7 (0.6–0.9)	*p* < 0.0005

* ANOVA and Kruskal-Wallis test; ** Chi ^2^ test; IQR (interquartile range); DM (diabetes mellitus); BMI (body mass index); PET (preeclampsia); HbA1C (haemoglobin A1C).

**Table 2:** Type of vascular complications in the studied groups at the beginning of pregnancy.

Vascular Complication	1993–2000 I *n* = 91	2001–2005 II *n* = 83	2006–2018 III *n* = 276	All *n* = 450	*p*-Value *
Number of vascular complications, *n* (%)	13 (14.3)	14 (16.7)	117 (41.0)	157 (34.9)	Gr.I vs. Gr.II *p* = 0.64 Gr.I vs. Gr.III *p* < 0.00001 Gr.II vs. Gr.III *p* < 0.00001
Background retinopathy, *n* (%)	25 (27.2)	20 (23.8)	35 (12.4)	80 (17.8)	Gr.I vs. Gr.II *p* = 0.61 Gr.I vs. Gr.III *p* < 0.0009 Gr.II vs. Gr.III *p* < 0.0114
Proliferative retinopathy, *n* (%)	13 (14.3)	17 (20.2)	57 (20.1)	87 (19.3)	Gr.I vs. Gr.II *p* = 0.28 Gr.I vs. Gr.III *p* = 0.18 Gr.II vs. Gr.III *p* = 0.97
Nephropathy, *n* (%)	7 (7.6)	7 (8.3)	9 (3.2)	23 (5.1)	Gr.I vs. Gr.II *p* = 0.86 Gr.I vs. Gr.III *p* = 0.06 Gr.II vs. Gr.III *p* < 0.04
Nephropathy and retinopathy, *n* (%)	33 (35.9)	26 (31.0)	59 (21.0)	118 (26.2)	Gr.I vs. Gr.II *p* = 0.81 Gr.I vs. Gr.III *p* < 0.0045 Gr.II vs. Gr.III *p* = 0.06
Renal transplantation, *n* (%)	1 (1.1)	0	1 (0.3)	2 (0.4)	Gr.I vs. Gr.III *p* = 0.41
Heart infarction, *n* (%)	0	0	1 (0.4)	1 (0.2)	
Nephropathy and heart infarction and retinopathy, *n* (%)	0	0	2 (0.7)	2 (0.4)	

* Chi ^2^ test.

**Table 3:** Characteristics of diabetes in studied subgroups at the beginning of observation concerning vascular complications.

Parameter	1993–2000 I *n* = 91	2001–2005 II *n* = 83	2006–2018 III *n* = 276	*p*-Value *
**Without Vascular Complications**				
Duration of diabetes, years (IQR)	15.0 (13.0–16.0)	18.0 (14.5–21.0)	17.0 (12.0–20.0)	*p* = 0.4
Age, years (IQR)	22.0 (21.0–24.0)	24.5 (23.0–27.0)	26.0 (23.0–25.2)	*p* = 0.07
Age at diagnosis, years (IQR)	8.0 (7.0–9.0)	7.5 (3.0–9.0)	9.0 (6.0–11.5)	*p* = 0.1
Initial pregnancy care, week (IQR)	12.5 (7.0–19.0)	7.5 (6.0–14.0)	8.0 (7.0–10.0)	*p* = 0.13
Mean HbA1C at the first visit, mmol/mol, (IQR)	60.7 (55.2–72.7)	45.4 (42.1–129.5)	59.6 (49.7–69.4)	*p* = 0.5
Mean HbA1C at the first visit, % (IQR)	7.7 (7.2–8.8)	6.3 (6.0–14.0)	7.6 (6.7–8.5)	*p* = 0.5
**Retinopathy**				
Duration of diabetes, years (IQR)	18.0 (16.0–21.0)	16.0 (13.5–20.5)	20.0 (15.0–23.4)	*p* = 0.17
Age, years (IQR)	29.0 (26.0–32.0)	27.0 (23.0–29.0)	29.2 (26.0–33.0)	*p* < 0.02
Age at diagnosis, years (IQR)	11.0 (6.0–13.0)	9.5 (8.0–12.0)	10.0 (6.0–12.0)	*p* = 0.2
Beginning of care in pregnancy, week (IQR)	9.0 (6.0–13.0)	9.5 (8.0–12.0)	8.0 (6.0–9.0)	*p* < 0.03
Mean HbA1C at the first visit, mmol/mol, (IQR)	60.7 (45.4–79.2)	55.2 (50.8–76.0)	58.5 (48.6–69.4)	*p* = 0.08
Mean HbA1C at the first visit, % (IQR)	7.7 (6.3–9.4)	7.2 (6.8–9.1)	7.5 (6.6–8.5)	*p* = 0.08
**Nephropathy**				
Duration of diabetes, years (IQR)	13.0 (12.0–17.0)	10.5 (9.0–13.0)	14.0 (13.7–18.0)	*p* = 0.08
Age, years (IQR)	24.0 (24.0–26.0)	31 (26.0–35.0)	31.0 (29.0–32.0)	*p* < 0.05
Age at diagnosis, years (IQR)	12.0 (9.0–13.0)	17.0 (17.0–23.0)	16.0 (11.0–18.0)	*p* < 0.02
Beginning of care in pregnancy, week (IQR)	9.0 (7.0–18.0)	5.5 (5.0–15.0)	6.0 (5.0–8.0)	*p* = 0.8
Mean HbA1C at the first visit, mmol/mol, (IQR)	61.7 (60.7–63.9)	48.6 (47.5–85.8)	53.0 (44.3–56.3)	*p* = 0.2
Mean HbA1C at the first visit, % (IQR)	7.8 (7.7–8.0)	6.6 (6.5–10.0)	7.0 (6.1–7.3)	*p* = 0.2
**Retinopathy and Nephropathy**				
Duration of diabetes, years (IQR)	18.0 (14.0–22.0)	14.5 (13.0–17.0)	18.0 (14.0–22.0)	*p* < 0.01
Age, years (IQR)	27.0 (24.0–31.0)	26.0 (23.0–30.0)	27.0 (24.0–31.0)	*p* = 0.3
Age at diagnosis, years (IQR)	10.0 (5.0–13.0)	10.0 (8.5–13.5)	10.0 (5.0–13.0)	*p* = 0.6
Beginning of care in pregnancy, week (IQR)	12.0 (8.0–15.0)	9.0 (7.0–18.0)	12.0 (8.0–15.0)	*p* < 0.002
Mean HbA1C at the first visit, mmol/mol, (IQR)	62.8 (46.4–70.5)	59.6 (53.6–74.9)	62.8 (46.4–70.5)	*p* = 0.8
Mean HbA1C at the first visit, % (IQR)	7.9 (6.4–8.6)	7.6 (7.05–9.0)	7.9 (6.4–8.6)	*p* = 0.8

* ANOVA and Kruskal-Wallis test; IQR (interquartile range); HbA1C (haemoglobin A1C).

**Table 4:** Perinatal outcome in the studied groups.

Parameter	1993–2000 I *n* = 91	2001–2005 II *n* = 83	2006–2018 III *n* = 276	All *n* = 450	*p*-Value **
Delivery (without miscarriages), week (SD) *	36.5 (2.5)	36.4 (3.0)	37.1 (2.1)	36.8 (2.4)	*p* = 0.06
Number of miscarriages ≤ 12 weeks, *n* (%) **	9 (9.9)	7 (8.4)	21 (7.6)	37 (8.2)	Gr.I vs. Gr.II *p* = 0.74 Gr.Ivs.Gr.III *p* = 0.50 Gr.II vs. Gr.III *p* = 0.80
Number of deliveries, *n* (%) **^,^^	82 (90.1)	75 (90.4)	231 (83.8)	388 (86.2)	Gr.I vs. Gr.II *p* = 0.91 Gr.I vs. Gr.III *p* = 0.15 Gr.II vs. Gr.III *p* = 0.15
Number of premature deliveries (22–36 weeks), *n* (%) **^,^^	34 (41.5)	25 (33.3)	59 (25.5)	118 (30.4)	Gr.I vs. Gr.II *p* = 0.31 Gr.I vs. Gr.III *p* < 0.01 Gr.II vs. Gr.III *p* = 0.1
Number of deliveries ≤ 32 weeks, *n* (%) **^,^^	4 (5.2)	8 (8.1)	7 (2.7)	19 (4.2)	Gr.I vs. Gr.II *p* < 0.02
Number of deliveries > 36 weeks, *n* (%) **^,^^	48 (58.5)	50 (66.7)	172 (74.5)	270 (69.6)	Gr.Ivs.Gr.II *p*=0.32 Gr.I vs. Gr. III *p* = 0.11 Gr.II vs. Gr.III *p* = 0.73
Number of spontaneous deliveries, *n* (%) **^,^^	2 (2.4)	10 (13.3)	35 (15.2)	46 (11.9)	Gr.I vs. Gr.II *p* < 00001 Gr.I vs. Gr.III *p* < 0.001 Gr.II vs. Gr.III *p* = 0.88
Number of cesarean sections, *n* (%) **^,^^	80 (97.6)	65 (86.7)	196 (71.0)	342 (88.1)	
Elective, *n* (%)	54 (67.5)	42 (63.6)	135 (68.9)	231 (67.5)	Gr.I. vs. Gr.II *p* = 0.71 Gr.I vs. Gr. III *p* < 0.01 Gr.II vs. Gr.III *p* = 0.52
Emergency (*n*, %)	22 (27.5)	11 (16.7)	22 (11.2)	55 (16.1)	
Mean newborn weight > 32 weeks, g (IQR) *	2950 (2420–3240)	3320 (2920–3700)	3290 (2940–3700)	3220 (2800–3640)	*p* < 0.0001
Newborns above 36 weeks ≥ 4000 g, *n* (%) **	3 (1.0)	6 (2.0)	34 (11.2)	43 (14.1)	Gr.I vs. Gr.II *p* = 0.24 Gr.I vs. Gr.III *p* < 0.01 Gr.II vs. Gr. III *p* = 0.13
Newborns above 36 weeks ≤ 2500 g, *n* (%) **	11 (3.6)	3 (1.0)	7 (2.3)	21 (6.9)	Gr.I vs. Gr.II *p* < 0.02 Gr.I vs. Gr.III *p* < 0.0005 Gr.II vs. Gr.III *p* < 0.0001
SGA, *n* (%) **	20 (24.4)	9 (12.0)	20 (8.7)	49 (12.6)	Gr.I vs. Gr.II *p* < 0.03 ^ Gr.I vs. Gr.III *p* < 0.002 ^ Gr.II vs. Gr.III *p* = 0.40 ^
LGA, *n* (%) **	5 (6.1)	16 (21.3)	50 (21.6)	71 (18.3)	Gr.I vs. Gr.II *p* < 0.01 ^ Gr.I vs. Gr.III *p* < 0.001 ^ Gr.II vs. Gr.III *p* = 0.95
APGAR score 1 min ≤ 7, *n* (%) **	45 (48.9)	23 (27.4)	32 (11.3)	100 (27.0)	Gr.I vs. Gr.II *p* < 0.03 Gr.I vs. Gr.III *p* < 0.0001 Gr.II vs. Gr.III *p* < 0.005
Umbilical artery pH, (IQR) *	7.21 (7.18–7.28)	7.26 (7.20–7.30)	7.24 (7.18–7.30)	7.24 (7.18–7.29)	*p* = 0.18
Umbilical artery pH ≤ 7.15, *n* (%) **	12 (13.0)	8 (9.5)	27 (10.5)	47 (10.2)	Gr.I vs. Gr.II *p* = 0.58 Gr.I vs. Gr.III *p* = 0.44 Gr.II vs. Gr.III *p* = 0.90
Umbilical artery pH < 7.05, *n* (%) **	2 (2.2)	3 (3.6)	10 (3.9)	15 (3.3)	Gr.I vs. Gr.II *p* < 0.03 Gr.I vs. Gr.III *p* < 0.0001 Gr.II vs. Gr. III *p* < 0.005
Number of malformations, *n* (%) **	3 (3.3)	2 (2.7)	9 (3.9)	14 (3.6)	Gr.I vs. Gr.II *p* = 0.72 Gr.I vs. Gr.III *p* = 0.9 Gr.II vs. Gr.III *p* = 0.62
Perinatal death until the 7th day, *n* (%) **	3 (3.3)	2 (2.7)	0	5 (1.1)	Gr.I vs. Gr.II *p* = 0.72 Gr.I vs. Gr.III *p* < 0.005 Gr.II vs. Gr.III *p* < 0.02

* ANOVA rang Kruskal-Wallis test; ** Chi ^2^ test; ^ number of patients whose data were available; SD (standard deviation); IQR (interquartile range); LGA (large for gestational age).

**Table 5:** Fetal outcome in relation to the vascular complications at the beginning of pregnancy in studied groups.

**Parameter**	**I** **1993–2000** ***n*** **= 91**	**II** **2001–2005** ***n*** **= 83**	**III** **2006–2018** ***n*** **= 276**	***p*** **-Value** *^,^^
**Without Vascular Complications**				
*n*	13	14	117	
Newborn weight, g (IQR) *	3100 (2800–3580)	3200 (3155–3795)	3470 (3080–4020)	*p* = 0.15
Delivery, week (IQR) *	38.0 (37.0–38.0)	38.0 (37.0–38.0)	38.0 (37.0–39.0)	*p* = 0.82
Delivery ≤ 32-week, *n* (%) **	0	0	21.7	Gr.II vs. Gr.III *p* = 0.64
SGA, *n* (%) **	1 (7.7)	3 (16.7)	6 (5.1)	Gr.I vs. Gr.II *p* = 0.31 Gr.I vs. Gr.III *p* = 0.70 Gr.II vs. Gr.III *p* < 0.02
Umbilical artery pH, (IQR) *	7.21 (7.18–7.25)	7.25 (7.19–7.29)	7.21 (7.12–7.28)	*p* = 0.38
APGAR score 1 min ≤ 7, *n* (%) **	3 (23.1)	3 (21.4)	12 (10.3)	Gr.Ivs.Gr.II *p*=0.92 Gr.I vs. Gr.III *p* < 0.003 Gr.II vs. Gr.III *p* = 0.17
**Retinopathy**				
*n*	38	37	92	
Newborn weight, g (SD) *	3160 (2200–3500)	3505 (2980–3810)	3290 (3000–3650)	*p* = 0.13
Delivery, week (SD) *	37.5 (36.0–38.0)	37.0 (36.5–38.0)	37.0 (36.0–38.0)	*p* = 0.90
Delivery ≤ 32-week, *n* (%) *	0	2 (5.4)	2 (2.2)	Gr.I vs. Gr.II *p* = 0.15 Gr.I vs. Gr.III *p* = 0.36 Gr.II vs. Gr.III *p* = 0.34
SGA, *n* (%) **	6 (15.8)	1 (2.7)	6 (6.5)	Gr.I vs. Gr.III *p* < 0.5 Gr.I vs. Gr.II *p* < 0.05
Umbilical artery pH, (IQR) *	7.25 (7.21–7.29)	7.30 (7.23–7.30)	7.27 (7.22–7.31)	*p* = 0.44
APGAR score 1 min ≤ 7, *n* (%) **	17 (44.7)	11 (29.7)	11 (11.9)	Gr.I vs. Gr.II *p* = 0.18 Gr.I vs. Gr.III *p* < 000001 Gr.II vs. Gr.III *p* < 0.02
**Nephropathy**				
*n*	8	7	9	
Newborn weight, g (IQR) *	2750 (1360–2800)	3200 (1500–3900)	2960 (2720–3330)	*p* = 0.09
Delivery, week (IQR) *	35.5 (31.0–37.0)	35.5 (34.0–39.0)	37.0 (36.0–38.0)	*p* = 0.68
Delivery ≤ 32-week, *n* (%) **	2 (25.0)	1 (14.3)	0	Gr.I vs. Gr.II *p* = 0.60 Gr.I vs. Gr.III *p* = 0.11 Gr.II vs. Gr.III *p* = 0.24
SGA, *n* (%) **	2 (25.0)	2 (28.6)	0	Gr.I vs. Gr.II *p* = 0.87 Gr.I vs. Gr.III *p* = 0.11 Gr.II vs. Gr.III *p* = 0.09
Umbilical artery pH, (IQR) *	7.19 (7.13–7.20)	7.26 (7.20–7.28))	7.16 (7.07–7.36)	*p* = 0.08
APGAR score 1 min ≤ 7, *n* (%) **	4 (50.0)	2 (28.6)	1 (11.1)	Gr.I vs. Gr.II *p* = 0.40 Gr.I vs. Gr.III *p* = 0.08 Gr.II vs. Gr.III *p* = 0.41
Perinatal death until the 7th day, *n* (%) **	0	1 (14.3)	0	*p* = 0.27
**Retinopathy and Nephropathy**				
*n*	33	26	59	
				
Newborn weight, g (IQR) *	2280 (1910–2900)	2645 (2090–3310)	2965 (2560–3290)	*p* < 0.03
Delivery, week (IQR) *	36.0 (34.0–37.0)	36.5 (35.0–38.0)	37.0 936.0–38.0)	*p* < 0.003
Delivery ≤ 32-week, *n* (%) **	2 (6.0)	2 (7.6)	2 (3.3)	Gr.I vs. Gr.II *p* < 0.80 Gr.I vs. Gr.III *p* = 0.55 Gr II vs. Gr.III *p* = 0.39
SGA, *n* (%) **	12 (36.3)	5 (19.2)	10 (16.9)	Gr.I vs. Gr.II *p* = 0.14 Gr.I vs. Gr.III *p* < 0.05 Gr II vs. Gr.III *p* = 0.80
Umbilical artery pH, (IQR) *	7.26 (7.18–7.29)	7.23 (7.19–7.27)	7.23 (7.15–7.30)	*p* = 0.78
APGAR score 1 min ≤ 7, *n* (%) **	21 (63.6)	7 (27.0)	7 (11.9)	Gr.I vs. Gr.II *p* < 0.05 Gr.I vs. Gr.III *p* < 0.00001 Gr II vs. Gr. III *p* = 0.08
Perinatal death until the 7th day (*n*, %) **	3 (9.1)	1 (3.8)	0 (0)	Gr.I vs. Gr.II *p* = 0.43 Gr.I vs. Gr.III *p* < 0.02 Gr.II vs. Gr.III *p* = 0.13

* ANOVA rang Kruskal-Wallis; ** Chi ^2^ test; IQR (interquartile range); SD (standard deviation); SGA (small for gestational age).

**Table 6:** Predictors of fetal outcomes in the study group.

Perinatal Outcome	Maternal Variable	OR	95% CI for Exp(B)		*p*-Value	R2 Nagelkerke
			Lower Limit	Upper Limit		
Preterm delivery < 37 completed weeks of gestation 88.8% of cases included in the model *p* for the model < 0.001	cohort II cohort III Age at DM diagnosis Maternal height Arterial hypertension	0.481 0.301 1.0630 960 2.110	0.228; 1.017 0.165; 0.572 1.019; 1.110 0.922; 0.999 1.133; 3.929		0.055 0.000 0.005 0.044 0.019	0.143
Emergency caesarean section 88.7% of cases included in the model *p* for the model = 0.004	cohort II cohort III Arterial hypertension	0.513 0.332 2.265	0.205; 1.284 0.160; 0.689 1.083; 4.739		0.154 0.003 0.030	0.074
Neonatal macrosomia (BW > 4000 g) 71.7% of cases included in the model *p* for the model = 0.041	cohort II cohort III Weight 0 BMI 0 RR diast. 0 HbA1C 0	1.525 2.614 1.100 0.798 0.985 1.344	0.221; 10.528 0.549; 12.442 1.015; 1.192 0.621; 1.027 0.944; 1.016 1.013; 1.782		0.669 0.227 0.020 0.079 0.474 0.040	0.110
Birth weight < 2500 g for term live births 80.1% of cases included in the model *p* for the model = 0.004	cohort II cohort III Weight 0 BMI 0 HbA1C 0	0.129 0.478 0.801 1.021 1.523	0.008; 2.182 0.090; 2.546 0.644; 0.996 0.599; 1.743 0.946; 2.452		0.156 0.387 0.046 0.938 0.083	0.281
1 min APGAR score ≤ 7 72.7% of cases included in the model *p* for the model < 0.001	cohort II cohort III early pregnancy diastolic BP	0.260 0.191 1.031	0.099; 0.679 0.101; 0.362 1.005; 1.057		0.006 0.000 0.019	0.194

CI (confidence interval); OR (odds ratio); DM (dibetes mellitus); BMI (body mass index); HbA1C (haemoglobin A1C); BP (blood pressure).

**Table 7:** Predictors of maternal outcomes in the study group.

Maternal Outcome	Maternal variable	OR	95% CI for Exp(B)		*p*-Value	R^2^ Nagelkerke
			Lower Limit	Upper Limit		
Gestational hypertension/ PET during pregnancy 100.0% of cases included in the model *p* for the model = 0.006	cohort II cohort III Arterial hypertension	2.685 1.732 0.247	1.027; 7.018 0.731; 4.105 0.075; 0.820		0.044 0.212 0.022	0.057

OR (odds ratio); PET (preeclampsia).

**Table 8:** Maternal parameters of renal function in early pregnancy as predictors of selected outcomes in the study group, independent from the model of treatment—data from the logistic regression models presents renal parameters which remained significant predictors for the outcomes after adjustment for the cohort.

The Outcome	Independent Predictors		
	GFR	Serum Creatinine Level	Daily Proteinuria (g/24 h)
BW > 4000 g	OR 1.021 (1.009; 1.034) *p* = 0.001		
BW < 2500 g	OR 0.976 (0.960; 0.992) *p* = 0.003	OR 42.19 (7.39; 240.99) *p* < 0.001	OR 2.141 (1.303; 3.517) *p* = 0.003
APGAR score ≤ 7			OR 1.65 (1.087; 2.505) *p* = 0.019
Premature delivery		OR 3.73 (1.14; 12.26) *p* = 0.030	
Maternal GWG above recommendations	OR 1.014 (1.003; 1.025) *p* = 0.013	OR 0.020 (0.001; 0.742) *p* = 0.034	

GFR (glomerular filtration rate); BW (birthweight); OR (odds ratio).

## References

[1] Ringholm L., Damm P., Mathiesen E.R. (2019). Improving pregnancy outcomes in women with diabetes mellitus: Modern management. Nat. Rev. Endocrinol..

[2] Mathiesen E.R., Ringholm L., Damm P. (2011). Pregnancy management of women with pregestational diabetes. Endocrinol. Metab. Clin. North. Am..

[3] Wender-Ozegowska E., Bomba-Opon D., Brazert J., Celewicz Z., Czajkowski K., Gutaj P., Malinowska-Polubiec A., Zawiejska A., Wielgoś M. (2018). Standards of Polish Society of Gynecologists and Obstetricians in management of women with diabetes. Ginekol. Pol..

[4] McElvy S.S., Demarini S., Miodovnik M., Khoury J.C., Rosenn B., Tsang R.C. (2001). Fetal weight and progression of diabetic retinopathy. Obstet. Gynecol..

[5] Klein B.E., Moss S.E., Klein R. (1990). Effect of pregnancy on progression of diabetic retinopathy. Diabetes Care.

[6] American Diabetes A. (2019). 14. Management of Diabetes in Pregnancy: Standards of Medical Care in Diabetes-2019. Diabetes Care.

[7] Landon M.B. (2007). Diabetic nephropathy and pregnancy. Clin. Obstet. Gynecol..

[8] How H.Y., Sibai B., Lindheimer M., Caritis S., Hauth J., Klebanoff M., MacPherson C., Van Dorsten P., Miodovnik M., Landon M. (2004). Is early-pregnancy proteinuria associated with an increased rate of preeclampsia in women with pregestational diabetes mellitus?. Am. J. Obstet. Gynecol..

[9] Miodovnik M., Rosenn B.M., Khoury J.C., Grigsby J.L., Siddiqi T.A. (1996). Does pregnancy increase the risk for development and progression of diabetic nephropathy?. Am. J. Obstet. Gynecol..

[10] Diabetes C., Complications Trial Research G. (2000). Effect of pregnancy on microvascular complications in the diabetes control and complications trial. The Diabetes Control and Complications Trial Research Group. Diabetes Care.

[11] Hod M., Van Dijk D.J., Weintraub N., Rabinerson D., Bar J., Peled Y., Erman A., Ovadia J. (1995). Diabetic nephropathy and pregnancy: The effect of ACE inhibitors prior to pregnancy on fetomaternal outcome. Nephrol. Dial. Transplant..

[12] Bar J., Chen R., Schoenfeld A., Orvieto R., Yahav J., Ben-Rafael Z., Hod M. (1999). Pregnancy outcome in patients with insulin dependent diabetes mellitus and diabetic nephropathy treated with ACE inhibitors before pregnancy. J. Pediatr. Endocrinol. Metab..

[13] Carr D.B., Koontz G.L., Gardella C., Holing E.V., Brateng D.A., Brown Z.A., Easterling T.R. (2006). Diabetic nephropathy in pregnancy: Suboptimal hypertensive control associated with preterm delivery. Am. J. Hypertens..

[14] Nathan D.M., Zinman B., Cleary P.A., Backlund J.Y.C., Genuth S., Miller R., Orchard T.J., Diabetes Control and Complications Trial/Epidemiology of Diabetes Interventions and Complications (DCCT/EDIC) Research Group (2009). Modern-day clinical course of type 1 diabetes mellitus after 30 years’ duration: The diabetes control and complications trial/epidemiology of diabetes interventions and complications and Pittsburgh epidemiology of diabetes complications experience (1983–2005). Arch. Intern. Med..

[15] Gadzinowski J., Kaliszewska-Drozdowska M.D., Kosinska M., Mazela J., Stoinska B. (2003). Birth weight and gestational age of newborns from Wielkopolski and Lubuski regions. Ginekol. Pol..

[16] Norgaard S.K., Vestgaard M.J., Jorgensen I.L., Ásbjörnsdóttir B., Ringholm L., McIntyre H.D., Damm P., Mathiesen E.R. (2018). Diastolic blood pressure is a potentially modifiable risk factor for preeclampsia in women with pre-existing diabetes. Diabetes Res. Clin. Pract..

[17] Nielsen L.R., Muller C., Damm P., Mathiesen E.R. (2006). Reduced prevalence of early preterm delivery in women with Type 1 diabetes and microalbuminuria—possible effect of early antihypertensive treatment during pregnancy. Diabet. Med. J. Br. Diabet. Assoc..

[18] Ekbom P., Damm P., Feldt-Rasmussen B., Feldt-Rasmussen U., Molvig J., Mathiesen E.R. (2001). Pregnancy outcome in type 1 diabetic women with microalbuminuria. Diabetes Care.

[19] PTD (2020). Zalecenia kliniczne dotyczące postępowania u chorych na cukrzycę 2020; Stanowisko Polskiego Towarzystwa Diabetologicznego. Diabetol. Prakt..

[20] Klemetti M.M., Laivuori H., Tikkanen M., Nuutila M., Hiilesmaa V., Teramo K. (2015). Obstetric and perinatal outcome in type 1 diabetes patients with diabetic nephropathy during 1988–2011. Diabetologia.

[21] Jensen D.M., Korsholm L., Ovesen P., Beck-Nielsen H., Moelsted-Pedersen L., Westergaard J.G., Moeller M., Damm P. (2009). Peri-conceptional A1C and risk of serious adverse pregnancy outcome in 933 women with type 1 diabetes. Diabetes Care.

[22] Kekäläinen P., Juuti M., Walle T., Laatikainen T. (2016). Pregnancy planning in type 1 diabetic women improves glycemic control and pregnancy outcomes. J. Matern.–Fetal Neonatal Med..

[23] Damm J.A., Asbjörnsdóttir B., Callesen N.F., Mathiesen J.M., Ringholm L., Pedersen B.W., Mathiesen E.R. (2013). Diabetic nephropathy and microalbuminuria in pregnant women with type 1 and type 2 diabetes: Prevalence, antihypertensive strategy, and pregnancy outcome. Diabetes Care.

[24] Reece E.A., Leguizamon G., Homko C. (1998). Pregnancy performance and outcomes associated with diabetic nephropathy. Am. J. Perinatol..

[25] Bullo M., Tschumi S., Bucher B.S., Bianchetti M.G., Simonetti G.D. (2012). Pregnancy outcome following exposure to angiotensin-converting enzyme inhibitors or angiotensin receptor antagonists: A systematic review. Hypertension.

[26] Temple R. (2011). Preconception care for women with diabetes: Is it effective and who should provide it?. Best Pract. Res. Clin. Obstet. Gynaecol..

[27] Polzlberger E., Hartmann B., Hafner E., Stumpflein I., Kirchengast S. (2017). Maternal Height And Pre-Pregnancy Weight Status Are Associated With Fetal Growth Patterns And Newborn Size. J. Biosoc. Sci..

[28] Nielsen L.R., Damm P., Mathiesen E.R. (2009). Improved pregnancy outcome in type 1 diabetic women with microalbuminuria or diabetic nephropathy: Effect of intensified antihypertensive therapy?. Diabetes Care.

[29] Spotti D. (2019). Pregnancy in women with diabetic nephropathy. J. Nephrol..

[30] Leach L., Taylor A., Sciota F. (2009). Vascular dysfunction in the diabetic placenta: Causes and consequences. J. Anat..

[31] Hanson U., Persson B. (1998). Epidemiology of pregnancy-induced hypertension and preeclampsia in type 1 (insulin-dependent) diabetic pregnancies in Sweden. Acta obstetricia et gynecologica Scandinavica.

[32] Gutaj P., Zawiejska A., Mantaj U., Wender-Ożegowska E. (2017). Determinants of preeclampsia in women with type 1 diabetes. Acta Diabetol..

[33] Hsu C.D., Hong S.F., Nickless N.A., Copel J.A. (1998). Glycosylated hemoglobin in insulin-dependent diabetes mellitus related to preeclampsia. Am. J. Perinatol..

